# Current models of care for disorders of sex development – results from an International survey of specialist centres

**DOI:** 10.1186/s13023-016-0534-8

**Published:** 2016-11-21

**Authors:** Andreas Kyriakou, Arianne Dessens, Jillian Bryce, Violeta Iotova, Anders Juul, Maciej Krawczynski, Agneta Nordenskjöld, Marta Rozas, Caroline Sanders, Olaf Hiort, S. Faisal Ahmed

**Affiliations:** 1 Developmental Endocrinology Research Group, School of Medicine, University of Glasgow, Zone 1, Office Block, RHC & QEUH Campus, 1345 Govan Road, Glasgow, G51 4TF UK; 2 Department of Child and Adolescent Psychiatry, Erasmus MC–Sophia Children’s Hospital, Rotterdam, The Netherlands; 3 Department of Paediatrics, Medical University of Varna, Varna, Bulgaria; 4 Department of Growth and Reproduction, Rigshospitalet, University of Copenhagen, Copenhagen, Denmark; 5 Department of Medical Genetics, Poznan University of Medical Science, Poznań, Poland; 6 Paediatric Surgery, Astrid Lindgren Children Hospital, Karolinska University Hospital, Stockholm, Sweden; 7 GrApSIA (Grupo de Apoyo al Síndrome de Insensibilidad a los Andrógenos), Barcelona, Spain; 8 University of Northern British Columbia, Canada & Adjunct Alder Hey Children Hospital, NHS Trust UK, Prince George, Canada; 9 Division of Experimental Paediatric Endocrinology and Diabetes, University of Lübeck, Lübeck, Germany

**Keywords:** Disorders of sex development, Rare diseases, Multidisciplinary team, Clinical networks

## Abstract

**Background:**

To explore the current models of practice in centres delivering specialist care for children with disorders of sex development (DSD), an international survey of 124 clinicians, identified through DSDnet and the I-DSD Registry, was performed in the last quarter of 2014.

**Results:**

A total of 78 (63 %) clinicians, in 75 centres, from 38 countries responded to the survey. A formal national network for managing DSD was reported to exist in 12 (32 %) countries. The paediatric specialists routinely involved in the initial evaluation of a newborn included: endocrinologist (99 %), surgeon/urologist (95 %), radiologist (93 %), neonatologist (91 %), clinical geneticist (81 %) and clinical psychologist (69 %). A team consisting of paediatric specialists in endocrinology, surgery/urology, clinical psychology, and nursing was only possible in 31 (41 %) centres. Of the 75 centres, 26 (35 %) kept only a local DSD registry and 40 (53 %) shared their data in a multicentre DSD registry. Attendance in local, national and international DSD-related educational programs was reported by 69, 78 and 84 % clinicians, respectively. Participation in audits/quality improvement exercises in DSD care was reported by 14 (19 %) centres. In addition to complex biochemistry and molecular genetic investigations, 40 clinicians (51 %) also had access to next generation sequencing. A genetic test was reported to be more preferable than biochemical tests for diagnosing 5-alpha reductase deficiency and 17-beta hydroxysteroid dehydrogenase 3 deficiency by 50 and 55 % clinicians, respectively.

**Conclusion:**

DSD centres report a high level of interaction at an international level, have access to specialist staff and are increasingly relying on molecular genetics for routine diagnostics. The quality of care provided by these centres locally requires further exploration.

## Background

Disorders of sex development (DSD) encompass a variety of different rare conditions that share a discordance in the typical chromosomal, gonadal and/or phenotypic sex. The consensus, reached in 2005, on the general principles of managing people with DSD represented a historic milestone for international and multidisciplinary collaboration in this area [1]. This consensus coincided with an increased global emphasis on the development of centres of expertise for rare conditions [2, 3]. Funding by an EU Seventh Framework Programme and a UK Medical Research Council grant supported the development of the International DSD Registry (I-DSD). This Registry has users from 50 countries from all 6 habitable continents. Of these countries, there are 59 centres that have entered almost 2500 cases [4]. More recently, the European Cooperation of Science and Technology (COST) Action DSDnet [5] supported the development of a network of clinicians, scientists and the affected community. Currently, 23 European countries have joined and, in partnership with 3 near neighbour and 5 international countries, are aiming to create a network to provide general information on DSD, and to give access to national specialist centres. It is anticipated that rare disease registries and forthcoming development of formal clinical and research networks will help establish a clinical framework for all centres of expertise by defining a standard of care as well as setting future research priorities in DSD [3].

A fundamental recommendation of the 2005 statement was that of a multidisciplinary team (MDT) approach to management of DSD, representing the new standard of care and support for children and their families [1, 6]. As a minimum standard, the specialist clinical MDT should include specialists in endocrinology, surgery and/or urology, clinical psychology/psychiatry, radiology, and nursing. In addition to delivery of clinical care, the MDT would have a responsibility to both educate healthcare staff outwith the MDT and to maintain the professional development of its own members through educational, audit and research activities [6].

The approach to investigating a newborn with a suspected DSD is likely to vary between centres and may be influenced by local availability and technological developments. Advances in biochemical analytical methods have led to an increase in the specificity and accuracy of measurement of steroid hormones and their metabolites in plasma and urine in the diagnostic work up of DSD [7–9]. Simultaneous developments in genetic and genomic technologies, combined with a marked reduction in costs, has provided a stimulus for change, with next-generation sequencing and whole-genome and -exome sequencing opening novel diagnostic strategies and expanding our knowledge of the underlying mechanisms of DSD [10].

With the advent of increasing international networks for rare conditions such as DSD, and the recent advances in diagnostics, there is a need to establish how closely specialist centres meet proposed standards including access to members of an MDT and local availability of desirable investigations. The aim of the study was to define the current models of multidisciplinary practice and to explore the diagnostic approach of clinicians delivering specialist care for children with DSD.

## Methods

### Design of survey

A working group of DSDnet conducted an international survey of centres/clinicians delivering specialist care for DSD. The working group consisted of professionals from paediatric endocrinology (AK, AJ, VI, OH, SFA), clinical psychology (AD), support group (MR), clinical genetics (MK), paediatric urology (AN), specialist nursing (CS) and project manager (JB). A preliminary version of the survey was piloted on clinicians of different clinical background and nationality to assess readability and time required for completion. The draft questionnaire was sent for review to all the members of the management committee of DSDnet. The final draft was reviewed and approved by all the group members. The duration of the survey was for two months between October 2014 and December 2014 and 124 clinicians working in the field of paediatric endocrinology, identified through the DSDnet management committee (website) as well as the registered clinical users of the I-DSD registry, were invited to participate (Table 1).

**Table 1:** The response rate to the questionnaire in each region per clinician, centre and country

	Sent			Responses					
Region	Clinicians	Centres	Countries	Clinicians		Centres		Countries	
	n	n	n	n	%	n	%	n	%
Europe	85	77	21	47	55	45	58	17	81
North America	7	7	2	5	71	5	71	2	100
South America	5	5	3	4	80	4	80	3	100
Africa	7	6	5	6	86	5	83	5	100
Asia & Australia	20	20	11	16	80	16	80	11	100
Total	124	115	42	78	63	75	65	38	91

The survey contained 17 items/questions divided into 2 sections. Items in the first section, directed to responding centres, included assessing MDT organisation and collaboration, networking, participation in clinical audit or quality improvement exercises, dissemination of knowledge, professional development, and the use of databases and data sharing. In the second section, directed to individual responding clinicians, participants were presented with the following clinical scenario: a newborn with palpable gonads in the upper inguinal region and genitalia that are so unusual in appearance that sex cannot be assigned at birth. The surveyed clinicians were then asked about their diagnostic approach, including specific details of the biochemical and genetic investigations they would perform in such circumstances and whether these tests were available locally in accredited clinical laboratories.

Analyses were conducted using IBM SPSS version 22 (SPSS Inc, Chicago). Variables are expressed as the value (percentage frequency). Comparison between groups, in which diagnostic and genetic tests are available or unavailable in a local accredited laboratory, was performed by *χ* ^2^ test or Fisher’s exact test. All tests were two-sided and P <0.05 was considered significant.

## Results

### Response rate and participants

A total of 124 clinicians from 115 centres in 42 countries were sent the questionnaire and the response rate to the questionnaire for clinician, centre and region is presented in Table 1. A total of 78 (63 %) clinicians from 75 (67 %) centres in 38 (91 %) countries responded to the survey. The respondent’s professional role was reported as paediatric endocrinologist in 70 (90 %), clinical geneticist in 4 (5 %), paediatrician in 2 (3 %), neonatologist in 1 (1 %) and adult endocrinologist in 1 (1 %). Of the 78 respondents, 68 (87 %) identified themselves as the clinical lead of the team that provided the clinical service for DSD in their centre.

### Formal national networks

A nationally organised formal network or a national plan for managing DSD was reported to exist in 12 (32 %) of the 38 countries. These countries were: Belgium (Belgisch Plan voor Zeldzame ziekten, Belgian-Luxembourg DSD network and registry), Brazil (DDSBrasil), Bulgaria (National Alliance of People with Rare Diseases), Finland (Finnish Research Network on Disorders of Sex Development), France (Centre de Référence Médico-Chirurgical des Maladies Rares du Développement et de la Différenciaton Sexuel), Germany (National Action Plan for Rare Diseases), Indonesia (Team Penyesuaian Kelamin/ Sexual Adjustment team), Japan (DSD committee of Japanese Society for Pediatric Endocrinology), Kuwait (Kuwait DSD network), Spain (Working Group on DSD of the Spanish Society for Paediatric Endocrinology), Sweden (Sveriges Nationella Nätverk för DSD) and Scotland within the UK (the Scottish DSD network).

### MDT organisation and collaboration

**Fig. 1:**
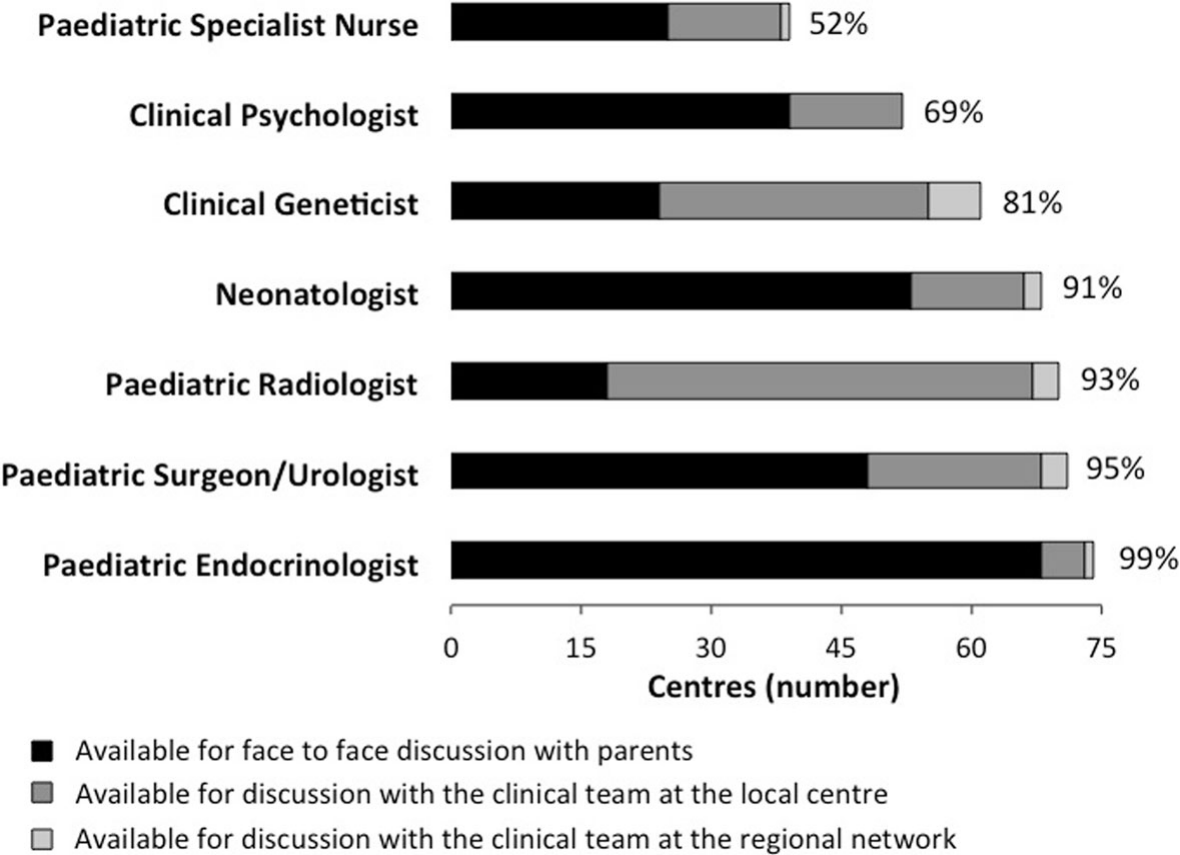
Individual paediatric specialist involvement in the initial evaluation of a newborn with suspected DSD in the 75 centres surveyed

**Fig. 2:**
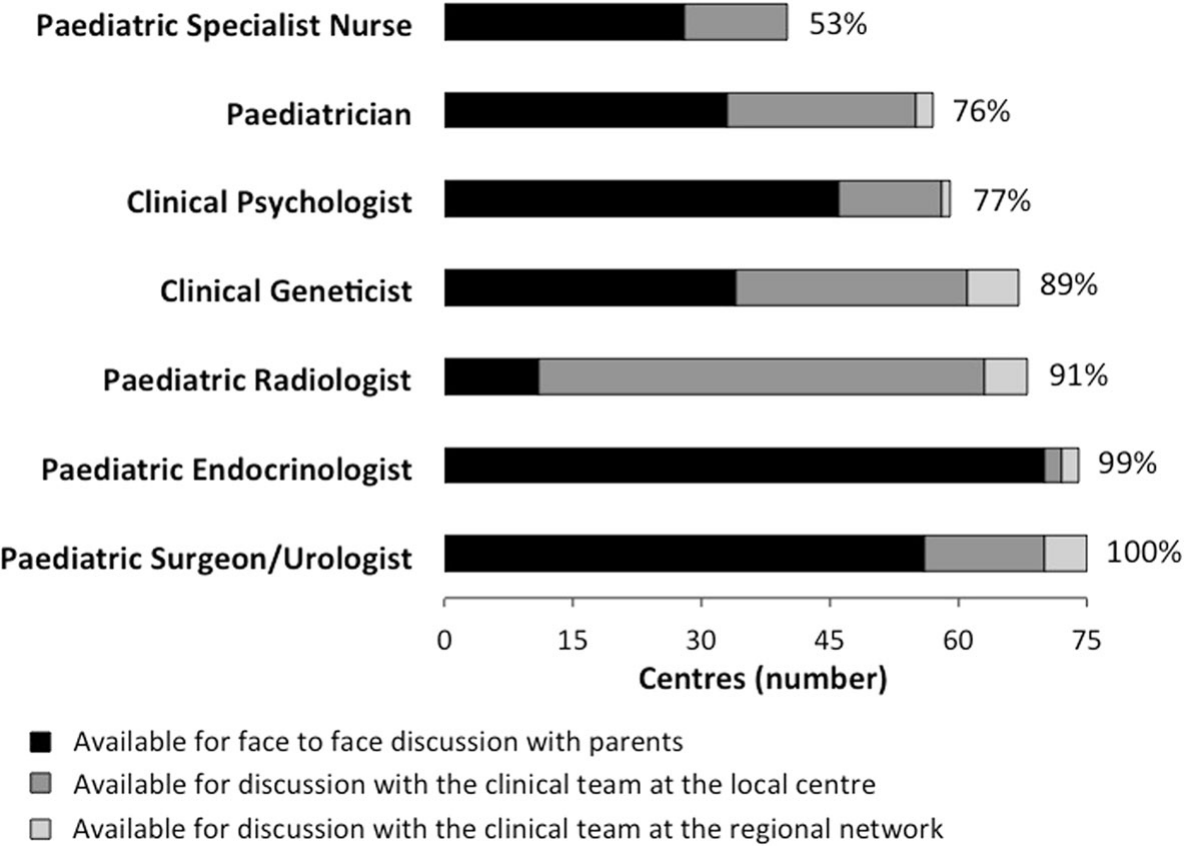
Individual paediatric specialist involvement during the first three months after presentation of a newborn with suspected DSD in the 75 centres surveyed

In 62 (83 %) of the 75 centres, the clinical lead of the MDT that provided DSD care was reported as a paediatric endocrinologist. The next commonest clinical lead was a clinical geneticist in 5 (7 %) centres. The paediatric specialists involved in the initial evaluation of a newborn with suspected DSD in the 75 centres are shown in Fig. 1. During the first week after presentation, a joint MDT assessment comprising paediatric specialists in endocrinology, surgery/urology, clinical psychology, and nursing, available in the same centre or as a part of a regional network, was possible in 31 (41 %) of the 75 centres. In the subsequent follow-up, over the first three months after the presentation of a newborn with suspected DSD, the paediatric specialists involved in the 75 centres are shown in Fig. 2. A team comprising of paediatric specialists in endocrinology, surgery/urology, clinical psychology, nursing and clinical genetics, available in the same centre or as a part of a regional network, was possible in 32 (43 %) centres. A paediatric specialist nurse was the commonest missing specialist from the MDT and was reported as desirable but not available in 22 (29 %) centres during initial evaluation and in 22 (29 %) centres during the following three months, while it was reported as not necessary in 14 (19 %) centres during the initial evaluation and in 13 (17 %) centres during the subsequent follow-up. Similarly, a clinical psychologist was desirable but not available in 18 (24 %) centres during initial evaluation and in 14 (19 %) centres during the next three months, while this requirement was reported as not necessary in 5 (7 %) centres during the initial evaluation and in 2 (3 %) centres during the subsequent follow-up. Links to a wider MDT consisting of specialists from adult endocrinology, gynaecology, biochemistry, social work and, to a clinical ethics forum was only possible in 6 (8 %) centres. Notably, 46 (61 %) centres reported the presence of a peer support group as desirable but not available in their region.

### Participation in registries, audits and quality improvement exercises

Of the 75 centres, 26 (35 %) reported that they only kept a local DSD registry, 40 (53 %) share their data in a multicentre, national or the International DSD registry and 9 centres (12 %) did not record any data. The main hurdles for participation in a registry were reported as lack of personnel by 48 centres (64 %), lack of available time by 42 centres (56 %) and difficulties in obtaining consent by 20 (27 %). Of the 75 centres*,* 14 (19 %) from 6 (16 %) countries reported that they participated in audit or quality improvement exercises in the field of DSD care.

### Professional development in DSD care

Of the 75 centres, 60 (80 %) were involved in organising meetings and case discussions, 42 (56 %) in involving students in research projects and 41 (55 %) in organising training days. Additional methods for disseminating knowledge in DSD included the invitation of health care professionals to participate in DSD clinics (26 centres, 35 %) and use of e-learning tools (13 centres, 17 %). Ten centres (13 %) reported no educational activities for engaging other health professionals. Of the 78 responding clinicians, 54 (69 %) had attended a local education event related to DSD within the year prior to the survey, the corresponding figures for attending a national or international event were 61 (78 %) and 64 (84 %), respectively. Of the 78 clinicians, 10 (13 %) attended only international educational programs and only 4 (5 %) did not attend any meeting.

### Selection & availability of endocrine & cytogenetic diagnostic tests

**Fig. 3:**
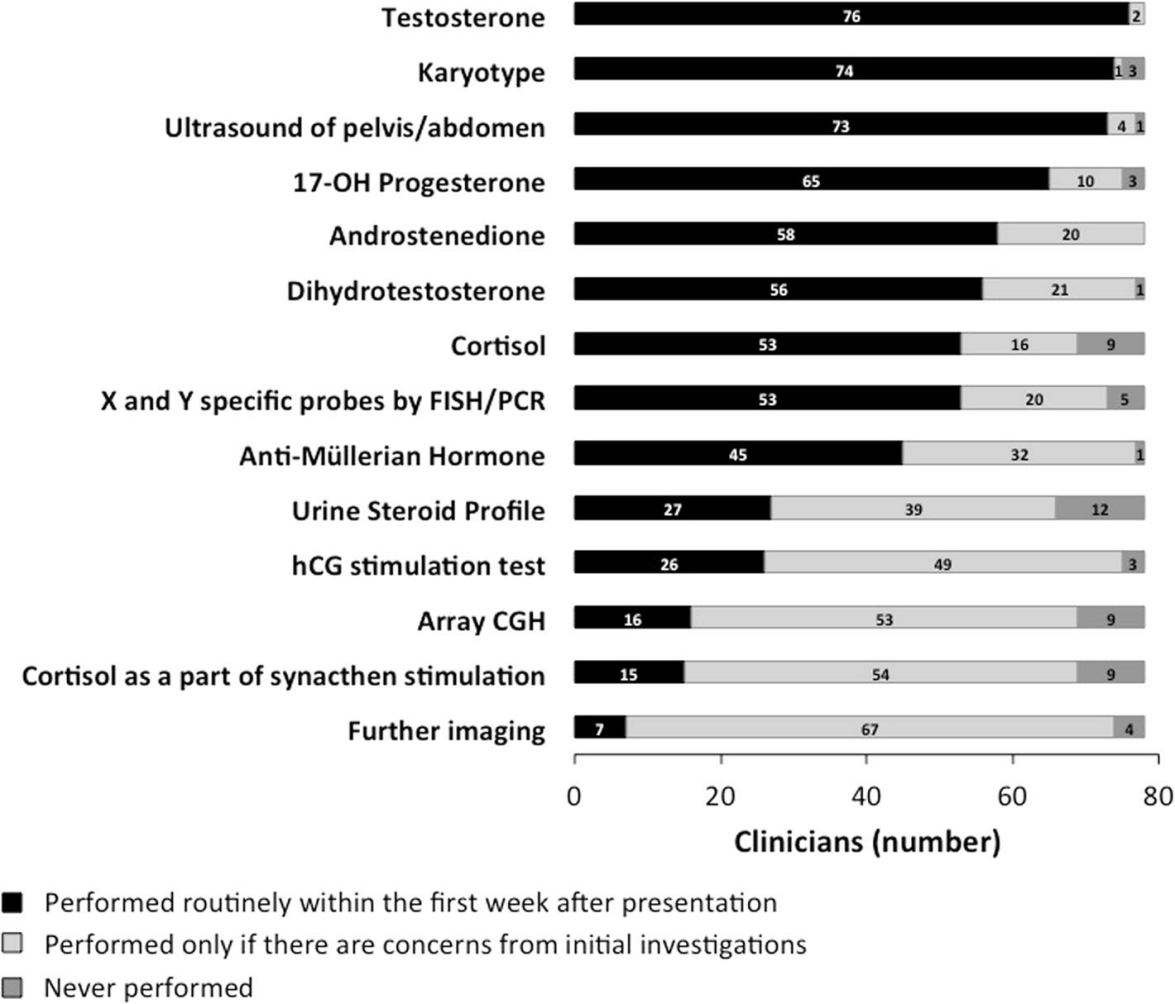
Selection preference for biochemical, cytogenetic, and imaging diagnostic tests of the 78 clinicians surveyed, at initial and subsequent follow-up, in a suspected case of 46,XY DSD. hCG, human chorionic gonadotropin; array CGH, array comparative genomic hybridisation; FISH, fluorescence in situ hybridisation; PCR, polymerase chain reaction

The investigations that clinicians would perform, at initial and subsequent follow-up, in a suspected case of 46,XY DSD are presented in Fig. 3. Of the 78 respondents, the commonest investigations that would be performed routinely, within the first week of presentation, included testosterone in 76 (97 %), karyotype in 74 (96 %), ultrasound of pelvis and abdomen in 73 (94 %), 17-hydroxyprogesterone in 65 (83 %), androstenedione in 58 (75 %), dihydrotestosterone (DHT) in 56 (73 %), cortisol in 53 (69 %), X and Y probes by fluorescence in situ hybridization (FISH) or polymerase chain reaction (PCR) in 53 (69 %) and anti-Müllerian hormone (AMH) in 45 (58 %).

We compared the selection of a diagnostic test on the basis of access to a locally accredited laboratory. A diagnostic test was reported to be available, in a locally accredited laboratory, when all tests were considered, in 85 % of the cases. In 60 % of those cases, with access to an accredited laboratory, the clinicians reported that they would select that test routinely and in 4 % the test would never be selected. In the 15 % of the cases, with no local accredited laboratory, only 30 % of the tests would be selected routinely and in 15 % the test would never be selected (*p* < 0.0001).

The most commonly unavailable diagnostic tests were urine steroid profile (n, 41, 53 %), array Comparative Genomic Hybridisation (CGH) (n, 21, 27 %), AMH (n, 16, 21 %) and DHT (n, 15, 19 %). When access to urine steroid profile was not available, 33 of 41 (81 %) clinicians would consider performing this test, if the test was available. The corresponding figures were 14 (67 %) for array CGH,16 (100 %) for AMH and 15 (100 %) for DHT.

### Selection & availability of molecular genetic tests

**Fig. 4:**
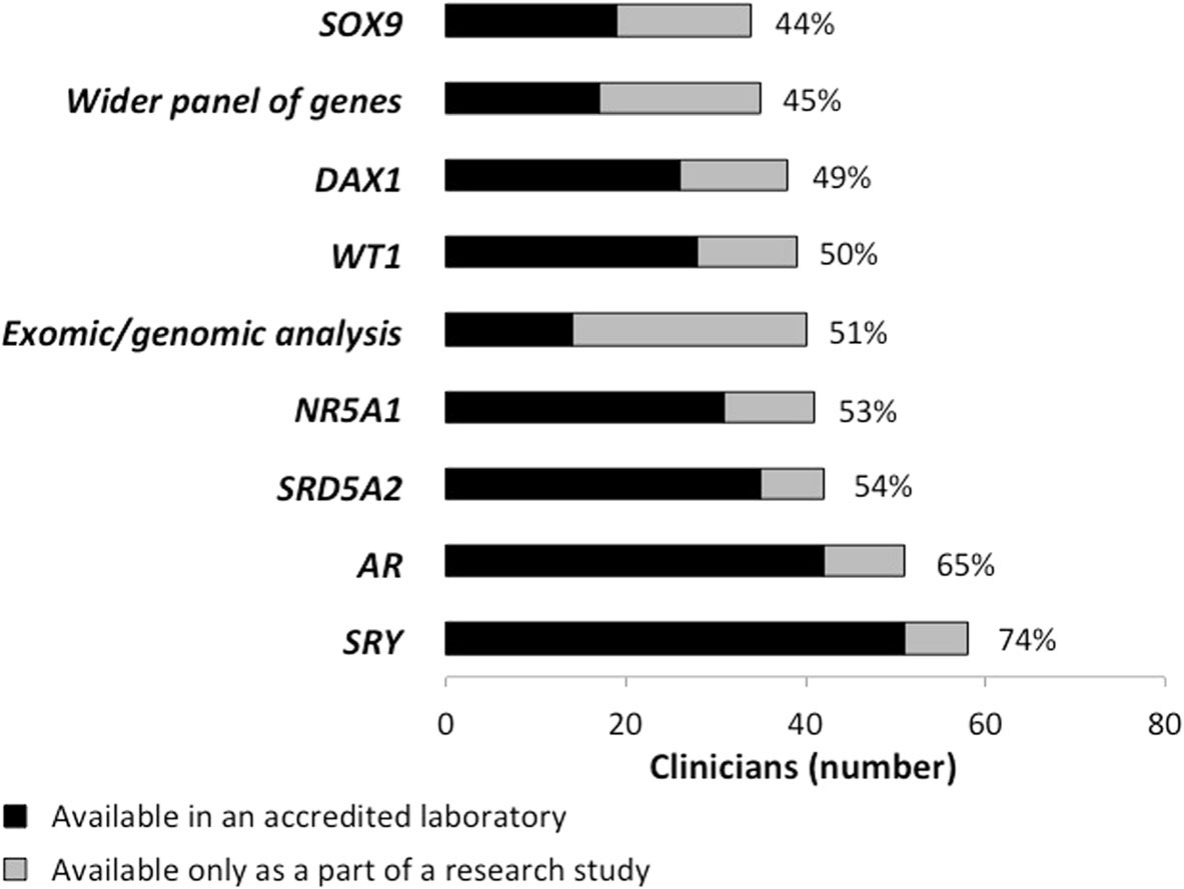
Local availability of individual genetic tests, either in accredited laboratories or as part of a research study, of 78 clinicians surveyed, both frequency and percentage

**Fig. 5:**
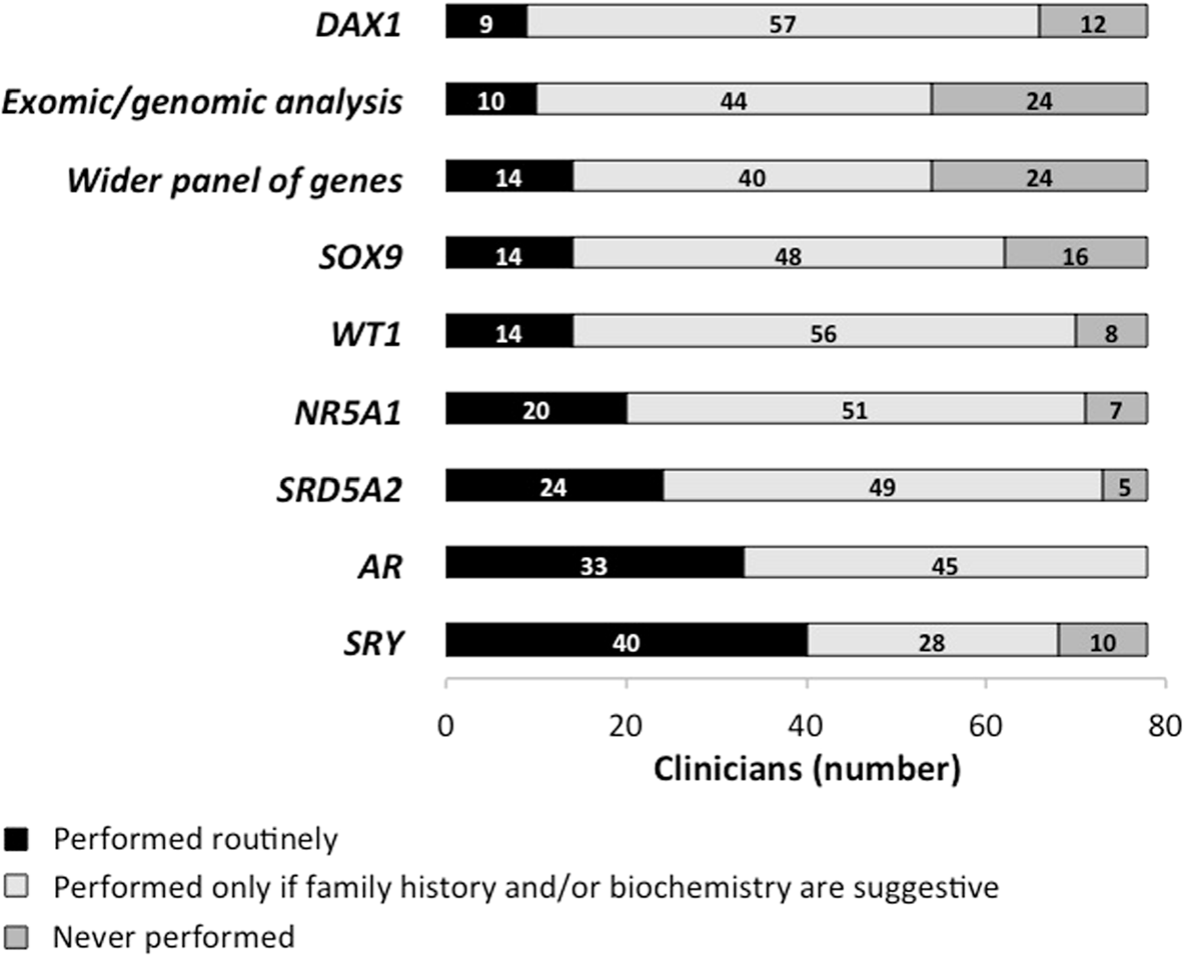
Selection preference for molecular genetic tests of the 78 clinicians surveyed, in the case of a newborn infant with 46,XY DSD

A majority of clinicians surveyed had access to a panel of genes commonly affected in XY DSD, however, in a large proportion this access was only available in research laboratories and not in clinically accredited laboratories (Fig. 4). Of the 78 clinicians, 62 (80 %) would perform routinely at least one genetic test (Fig. 5). The most common genetic tests that clinicians would perform routinely in a case of 46,XY DSD included *SRY* in 40 (51 %), *AR* in 33 (43 %), *SRD5A2* in 24 (31 %) and *NR5A1 in 20* (26 %). It was also noted that 14 (18 %) and 10 (13 %) would routinely consider performing a wider panel of genes and exomic/genomic analysis respectively. In addition, if family history and/or biochemistry were suggestive, clinicians would check *DAX1* in 57 (73 %), *WT1* in 56 (71 %), *NR5A1* in 51 (65 %), *SRD5A2* in 49 (62 %) and *SOX9* in 48 (61 %). Conversely, 24 (31 %) clinicians reported that they would never perform exomic/genomic analysis and 24 (31 %) would never perform a wider panel of genes in reference to the same case of 46,XY DSD.

We compared the selection of a genetic test on the basis of access to a locally accredited laboratory. Overall, clinicians reported access to a genetic test in a local accredited laboratory in 38 %. Presented with a case of a newborn with 46,XY DSD and a locally available genetic test in an accredited laboratory, in 95 % of the cases the clinicians would select this test as matter of routine or if family history and/or biochemistry were suggestive of a diagnosis. Clinicians reported a lack of local availability to a genetic test in 62 % of cases and, in 22 % of these cases the clinicians would never consider performing this test (*p* < 0.0001).

### Condition specific preference of investigations in 46,XY DSD

The surveyed clinicians were asked to choose between molecular genetic and biochemical confirmation for diagnosing a case of 5α reductase deficiency and a case of 17β hydroxysteroid dehydrogenase 3 (17βHSD3) deficiency. For 5α reductase deficiency, 39 (50 %) clinicians selected genetic testing; 29 (37 %) selected testosterone: DHT ratio; and 10 (13 %) selected urine steroid profile as the single most preferable test in diagnosis. For diagnosing 17βHSD3 deficiency, 43 (55 %) clinicians selected genetic testing; 25 (32 %) selected testosterone: DHT ratio; and 10 (13 %) selected urine steroid profile as the single most useful test in diagnosis.

## Discussion

The aim of this study was to explore the current models of care of clinicians working in specialist centres for DSD. The response rate to the survey was good, and the respondents represented centres from all continents and with different financial and cultural backgrounds.

Our study showed that the majority of centres implemented an MDT, allowing the possibility for families to be supported and make informed decisions at a crucial time. A previous study of 60 DSD centres in 23 countries, reported close to full cover, in 58 % of centres, from those subspecialties recommended by the 2005 Consensus [1], whilst only 7 % of centres were missing a key service, that is, paediatric endocrinologist, urologist/surgeon and/or psychological services, suggesting that the multidisciplinary approach to DSD had been successfully implemented across Europe [11]. A major difference from our study was that the previous study asked about the presence of specialists involved at any stage of the child’s clinical journey and not specifically at the time of initial approach or proceeding three months. Our study showed that only 40 % of the centres had an MDT inclusive of paediatric specialists in endocrinology, surgery/urology, clinical psychology, and nursing available during the initial approach, and only 40 % had a joint team consisting of paediatric specialists in endocrinology, surgery/urology, clinical psychology, nursing and clinical genetics during the proceeding three months. Most commonly, a specialist nurse and psychologist were missing from the MDT, and conversely the majority of clinicians reported that they would wish involvement of a nurse or psychologist in those circumstances where they were unavailable. It can be inferred that the majority of clinicians understand the importance of all denoted key members of the MDT, and without imposed financial constraints would wish to design a service that had capacity to offer the benefits of holistic care to any given family [12].

The 2005 Consensus and, more recently, the EU criteria for centres of expertise, have highlighted the need for the creation and maintenance of a database for rare conditions such as DSD [2, 13]. Such databases exist in many regional and national centres, as 90 % of the centres in this study reported keeping a DSD database, and to date have provided invaluable insight into several aspects of DSD including epidemiology [14, 15], aetiology [16], disease expression [17] and long-term outcome [18, 19]. In this study, half of the centres reported sharing their data in a multicentre registry. It is possible that the true participation in such registries is much lower as clinicians invited to complete the survey were in part identified through the I-DSD registry. The reported barriers to using a registry included time and personnel support and these are areas that will need to be addressed. Whilst consent was reported as another barrier, preliminary data from a survey of service users in one centre did not raise this as an important issue [20].

Most centres surveyed reported a lack of participation in formal clinical audit. Assessing outcome, patient satisfaction and quality of care provided, i.e. not only an expression of which specialists and at what time, is inherently more difficult, and requires participation in internal and external quality schemes which may in turn produce quality of care indicators and implement outcome measures [2, 21, 22]. It is possible that some centres may have been involved in audit activities targeted at laboratory processes but these were not captured. To maintain expertise of rare diseases, clinicians should engage in continuous professional development through attendance at specialist meetings and discussion of complex cases with international colleagues. Presently, maintenance of an individual clinician’s educational needs often requires attendance at international conferences.

This study confirmed that local access to specialist biochemical and genetic tests influences the diagnostic process. It has been shown previously that in many Western European countries, such as UK, Germany and France, the use of national networks allowed for timely provision of almost all biochemical and genetic tests, while in other countries, many tests are performed in private laboratories or through international collaboration, with long turnaround times [23]. The rationale for investigating a newborn with a suspected DSD may include the need to determine the sex of rearing, to anticipate early medical problems, to explain the aetiology of DSD and to develop a long-term management plan. The current initial approach of most surveyed clinicians is to utilise those investigations which offer additional phenotypic information, and would include urgent endocrine testing (testosterone, 17-hydroxyprogesterone, cortisol) imaging studies (pelvic ultrasound), and rapid identification of the sex chromosome complement by karyotype analysis or FISH/PCR with X and Y probes. In those cases where investigations were not available locally, commonly urine steroid profiles, AMH or array CGH, there was greater disparity in whether these investigations were offered as first or second line.

Molecular genetic testing, either of single candidate genes or a gene panel, is increasingly common in specialist centres, as approximately 80 % of clinicians would perform genetic testing as routine in a newborn with suspected 46,XY DSD and 50 % of them would select genetic testing as the single most preferable method for diagnosis. As new genomic technologies have rapidly become an integral part of the diagnostic armoury in the field of DSD, a suggested alternative diagnostic approach would make use of next generation sequencing (NGS) as first-line [10, 24]. Notably, nearly one-fifth of the surveyed clinicians reported ready availability and preference to this approach. However, this approach may face hurdles including long turnaround times, high costs, and difficulties in the interpretation of the results. These obstacles are likely to be overcome in the future, and NGS is likely to become the mainstay of investigations in diagnosis of DSD. Although, the majority of the respondents to this survey suggested that genetic testing should be targeted and based on history and biochemical characteristics, it was interesting to note that the majority of respondents believed that a genetic confirmation of a diagnosis was preferable to a biochemical confirmation when encountering a case of 5α reductase deficiency or 17βHSD3 deficiency, highlighting the shift towards molecular genetics and an appreciation of the lack of sensitivity in arbitrary metabolite ratios in diagnosing these conditions [25–27].

Differences in the composition of the MDT as well as diagnostic tests have been highlighted by others [23] and will be influenced by local medical, financial, geographic, or personal reasons. A model for delivery of education should be analogous to that of clinical support offered to local centres. In the best examples provided, regional centres are responsible for disseminating knowledge to local centres through opportunities for clinical meetings, case discussion and observation in regional DSD clinics; organising conferences and training days accessible to all health care professionals; and use of e-learning tools. Whilst a number of centres were involved in continuous professional development, there is clearly a need to explore other models. Unlike clinical support, it is possible that the use of remote technology may lead to wider involvement in educational events.

Approximately one third of countries or regions surveyed, had attempted to overcome these hurdles with the development of managed clinical networks for rare conditions such as DSD. The remit of a clinical network should be to ensure the provision of an equitable state of-the-art service for all affected children and adolescents in a region through formal structured referral pathways. A network also facilitates the creation of protocols, with consideration for local and national availability of services, setting and monitoring of national standards of care, rational utilisation of other services such as clinical genetics and clinical biochemistry and provides a forum for education and professional development. Research and audit are vital for the management of DSD, and clinical networks have a strong potential to drive these activities with the development of care standards including patient experience data and peer-observation of clinical care provision [6, 28–30]. In cases where there is uncertainty at a regional level, there is a need to create a worldwide network of experts and stakeholders in DSD, linking existing national and regional networks throughout the world.

There are limitations inherent in a questionnaire-based study due to the nature of data collection and potential response bias. Thirty-seven percent of clinicians invited to participate did not respond to the survey and, therefore, it is possible that response rates were higher among centres with practices more compliant with the proposed standards of care and would effectively produce a positive bias in our report. Gathering more detailed information on each centre may have been advantageous if we wished to further delineate those factors, which were associated with response, but a balance needed to be struck between maximising data collection and achieving a desirable response rate. Overall, there was international participation to the survey, however some countries were relatively over-represented (UK, Germany, Bulgaria, Spain, Turkey) and participating centres within countries did not provide uniform coverage to all geographical areas. It is possible that practice and institutional characteristics vary between regions and countries, factors which may influence results of the study.

## Conclusion

In summary, we report the findings of a large, international survey of DSD specialist centres, reflecting contemporary clinical practice in DSD. An increasing number of DSD centres have access to specialist staff, however a gap still exists between the current models of clinical care and that of the ideal comprehensive MDT. We have focussed here on the clinical infrastructure, however the actual delivery and quality of care provided in the MDT described requires further exploration through both clinical audit and individual user feedback. We have shown a considerable variation in the diagnostic evaluation of a newborn with suspected DSD, an apparent shift towards molecular genetic testing, and have provided evidence that access to specialist tests influences the diagnostic process. Collaboration through a network of specialist centres could assist in bridging gaps in access to expert clinicians and other key members of the MDT and diagnostic investigations for DSD. With the emergence of a new era of medical management that demands collaborative and whole-systems treatment for these complex conditions, the medical community in partnership with individuals with DSD, families and support groups has a unique opportunity to move to the next phase of developing measurable standards of DSD care.

## Abbreviations

17beta-HSD3: 17β hydroxysteroid dehydrogenase 3
AMH: Anti-müllerian hormone
array CGH: Array comparative genomic hybridisation
DHT: Dihydrotestosterone
DSD: Disorders of sex development
FISH: Fluorescence in situ hybridization
MDT: Multidisciplinary team
NGS: Next generation sequencing
PCR: Polymerase chain reaction
